# Lower Respiratory Tract Bacterial Profiles Are Associated With Respiratory Severity and Bronchopulmonary Dysplasia in Neonates

**DOI:** 10.1002/ppul.71364

**Published:** 2025-11-11

**Authors:** Jelte Kelchtermans, Pelton Phinizy, Joseph Piccione, Sharon A. McGrath‐Morrow

**Affiliations:** ^1^ Children's Hospital of Philadelphia Philadelphia Pennsylvania USA; ^2^ Perelman School of Medicine University of Pennsylvania Philadelphia Pennsylvania USA

**Keywords:** bronchoscopy, *Klebsiella pneumoniae*, microbiology, prematurity

## Abstract

**Introduction:**

Bronchopulmonary dysplasia (BPD) is a major complication of prematurity, marked by heterogeneous pulmonary phenotypes and variable clinical outcomes. The airway microbiome may influence disease severity and progression, yet quantitative associations between airway pathogens and clinically relevant outcomes remain poorly understood.

**Methods:**

We conducted a retrospective analysis of 204 neonates who underwent flexible bronchoscopy with quantitative bronchoalveolar lavage (BAL) cultures in the NICU at the Children's Hospital of Philadelphia. Cultures yielding ≥ 10,000 colony‐forming units per milliliter for a single bacterial species were classified as positive. Respiratory severity score (RSS), calculated as the product of mean airway pressure and fraction of inspired oxygen, served as the primary indicator of respiratory status. Linear, logistic, and negative binomial regression models were used to assess associations between bacterial species and clinical outcomes, adjusted for sex and race, with standard errors clustered at the patient level.

**Results:**

No bacterial species were significantly associated with RSS after correction for multiple testing. *Klebsiella pneumoniae* was associated with a diagnosis of BPD (adjusted *p* = 0.026), but no organisms were significantly associated with prolonged time to extubation. In secondary analyses, the presence of several organisms was significantly associated with higher MAP, including *K. pneumoniae* (*β* = 2.66, FDR‐adjusted *p* = 0.014).

**Conclusions:**

Multiple bacterial species identified on quantitative BAL culture were associated with higher mean airway pressure, and *K. pneumoniae* was additionally associated with BPD diagnosis. These findings support the potential utility of quantitative microbiologic data in risk stratification and management of neonatal respiratory disease.

## 1 Introduction

Globally, prematurity remains a leading cause of morbidity and mortality in children under 5 years of age [1]. Among its most serious complications is bronchopulmonary dysplasia (BPD), a chronic lung disease that contributes significantly to long‐term respiratory and neurodevelopmental impairment [2, 3]. Despite decades of research, the factors driving variability in respiratory outcomes and development of BPD among children born prematurely remain incompletely understood [3].

BPD itself is increasingly recognized not as a single disease entity, but as a syndrome encompassing diverse pulmonary phenotypes, with varying degrees of airway inflammation, alveolar simplification, pulmonary vascular disease, and abnormal gas exchange [3, 4]. While this clinical heterogeneity complicates both prognostication and management, it suggests that for some patients additional, potentially modifiable, contributors to disease progression remain undetermined.

One longstanding area of interest is the role of infection and airway colonization in shaping pulmonary outcomes [5]. Even with modern advances in respiratory care, the preterm lung remains highly susceptible to injury from barotrauma and oxidative stress, with these insults known to compromise key host defenses including the epithelial barrier, mucociliary clearance, and immune cell proliferation [5, 6]. Perhaps unsurprisingly, early studies linked Gram‐negative colonization of the lung to increased severity of lung injury and BPD [5].

More recently, research into the neonatal airway microbiome has added further complexity to this picture. Rather than being sterile at birth, the airways of preterm infants appear to harbor distinct microbial communities that evolve over the first weeks of life, shaped by factors such as delivery mode, antibiotic exposure, respiratory support, and feeding practices [7, 8]. Several studies have identified decreased bacterial diversity, increased abundance of Ureaplasma or Klebsiella species, and reduced presence of commensal organisms like Lactobacillus in infants who go on to develop BPD [9].

While research on the preterm airway microbiome has expanded in recent years, most studies to date have been limited by small sample sizes, lack of clinical correlation, and substantial heterogeneity in both methodology and patient populations. Moreover, neonatal care itself is continually evolving, with ongoing changes in ventilation strategies, antibiotic stewardship practices, and use of postnatal steroids, all of which may influence patterns of airway colonization and inflammation [8]. Compounding this, the hospital microbiome itself has shifted over time, with increased prevalence of resilient and highly pathogenic organisms such as hypervirulent strains of *Klebsiella pneumoniae* [10]. Once primarily associated with infections in immunocompromised adults, *K. pneumoniae* is now frequently isolated in neonatal intensive care settings, where it can colonize respiratory equipment, form biofilms, and evade immune responses through a combination of hypercapsule production, siderophore‐mediated iron scavenging, and antibiotic resistance [10]. These emerging strains have the potential to alter both the dynamics of airway colonization and the inflammatory milieu of the developing lung, yet their clinical significance in neonatal respiratory disease remains poorly defined.

One promising recent development in the effort to quantify respiratory illness severity in preterm infants is the respiratory severity score (RSS), a simple metric calculated as the product of mean airway pressure (MAP) and fraction of inspired oxygen (FiO2) [11]. Originally developed as a surrogate for oxygenation efficiency in intubated neonates, the RSS has been shown to predict both short‐ and long‐term respiratory outcomes, including duration of mechanical ventilation and risk of developing BPD [12]. However, it remains unknown whether variation in airway microbiology, particularly the presence of potentially pathogenic bacteria identified on quantitative culture, correlates with increased RSS or worse clinical trajectories.

Given the potential role of infection and dysbiosis in the pathogenesis of BPD, there is a critical need to bridge the gap between microbiologic findings and clinically meaningful measures of disease severity. In this study, we sought to evaluate the relationship between airway bacterial burden and clinical measures of respiratory illness in a contemporary cohort of NICU patients undergoing diagnostic bronchoscopy. Although bronchoalveolar lavage (BAL) and protected specimen brush techniques have long been used in adults to obtain lower airway samples while minimizing upper airway contamination, their application in neonates was historically limited by the small diameter of neonatal endotracheal tubes [13]. However, the advent of increasingly miniaturized flexible bronchoscopes over the past decade has made safe and effective lower airway sampling feasible even in very small infants. Leveraging this capability, we used quantitative BAL culture data to examine whether the presence of specific bacterial species was associated with greater respiratory severity, prolonged time to extubation, or increased risk of BPD. By linking quantitative microbiologic data to clinical endpoints, our goal was to clarify the potential relevance of BAL culture results for prognostication and management in neonatal respiratory disease.

## 2 Methods

### 2.1 Population

We extracted data from the Children's Hospital of Philadelphia (CHOP) electronic medical record (EMR) for all flexible bronchoscopies performed in the neonatal intensive care unit (NICU), as documented in the associated procedure notes. To facilitate data extraction, only bronchoscopies performed after laboratory data migration (April 2016) to the current Epic‐based EMR system were included. Patients were included if results from at least one quantitative respiratory culture were available. For all included patients, we collected the following variables: diagnosis of BPD as documented in the EMR, sex, race as recorded in the EMR, gestational age, age at the time of bronchoscopy, number of days since intubation, and number of days from bronchoscopy to extubation (defined as the absence of an invasive airway documented in the EMR for at least two consecutive calendar days). Additionally, we recorded the FiO2 and MAP on the day of bronchoscopy. Records missing gestational age were excluded from analysis. This study protocol was reviewed and classified as Exempt from IRB review and informed consent under category four by the CHOP IRB (IRB protocol 24‐022076).

### 2.2 Respiratory Culture Interpretation

To identify the most relevant organisms for analysis, we first conducted a comprehensive review of all bronchoscopy‐derived respiratory culture results available at our center. A culture was considered positive for a given organism if the species name was explicitly mentioned in the result string and reported at a concentration of ≥10,000 colony‐forming units per milliliter (CFU/mL) for each bacterial species isolated. Cultures were categorized as “normal flora” if the final culture result indicated “normal respiratory flora present,” “no growth,” or similar wording.

### 2.3 RSS

For patients who were intubated for at least 1 day before bronchoscopy, we calculated the RSS as the product of the MAP and the FiO2 [11]. To minimize potential bias from the bronchoscopy procedure itself, such as transient increases in support in anticipation of the procedure or post‐procedural instability, we used the average of the first three recorded MAP and FiO2 values on the day of bronchoscopy. These measurements were typically recorded between midnight and early morning. Additionally we included two safegards to avoid including values impacted by the procedure. First, when a procedural time‐out was documented, we excluded any data starting 1 h before that time stamp. Second, when no time‐out was recorded, we excluded all data after 10 a.m., as procedures before that time are exceedingly rare due to morning rounds.

### 2.4 Statistical Analysis

For all organisms identified at least 15 times we tested whether specific bacterial species were associated with RSS, time to extubation, and BPD. For each organism, we compared infants with ≥10,000 CFU/mL of the organism to those with normal flora. Linear regression models were used to examine relationships between positive cultures and RSS, negative binomial regression models were used to assess whether bacterial presence was associated with the number of days from bronchoscopy to extubation, and logistic regression models were used to evaluate associations between bacterial species and the diagnosis of BPD. All models were adjusted for sex and race, with robust standard errors clustered by patient to account for repeated measures [14, 15]. All analyses were performed in R (version 4.4.1). *p* values were corrected for multiple testing using the Benjamini‐Hochberg false discovery rate (FDR) method to account for the four organisms examined. Because RSS is a composite measure that incorporates both MAP and FiO2, we also evaluated the two components individually in secondary analyses. This was prompted by additional safeguards described above, which revealed greater variability in the FiO₂ component. As a result, MAP demonstrated a more stable and robust association with bacterial pathogens, and results for MAP are therefore reported alongside the prespecified RSS analyses. Of note, when analyzing time to extubation, we excluded patients that died or required tracheostomy before extubation.

## 3 Results

### 3.1 Population

The study included 204 neonatal patients, of whom 73 (35.8%) had a diagnosis of BPD and 131 (64.2%) did not (As shown in Table 1). The overall cohort was predominantly male (58.8%), with males comprising 53.4% of the BPD group and 61.8% of the non‐BPD group (*p* = 0.307). The average number of bronchoscopies performed per patient was similar between groups, with a mean of 1.32 ± 0.57 in the BPD group and 1.34 ± 0.72 in the non‐BPD group (*p* = 0.832). Among patients without BPD, race was recorded as “Black or African American” for 41.2% of patients, “White” for 32.1%, “Other or not available” for 22.1%, and “Asian” for 4.6%. In the BPD group, race was recorded as “Black or African American” for 52.1% of patients, “White” for 30.1%, and “Other or not available” for 17.8%; no patients with BPD identified as Asian. These distributions did not differ significantly between groups (*p* = 0.168). Patients with BPD were born significantly more premature, with a mean gestational age of 181.0 ± 13.6 days compared to 212.0 ± 39.5 days in those without BPD (*p* < 0.001). Of note, three children who died (9.1%) had *Proteus mirabilis* identified on at least one culture, compared with 2 surviving children (1.2%; *p* = 0.038). Because of the very small numbers, we did not attempt adjusted analyses, as any regression model would be unstable and prone to overfitting.

**Table 1:** Population overview.

	No BPD	BPD	*p* value
Number of patients	131	73	
Number of recorded bronchoscopies per patient (mean [SD])	1.34 (0.72)	1.32 (0.57)	0.832
Sex = male (%)	81 (61.8)	39 (53.4)	0.307
Recorded race (%)			0.168
Asian	6 (4.6)	0 (0.0)	
Black or African American	54 (41.2)	38 (52.1)	
White	42 (32.1)	22 (30.1)	
Other or not recorded	29 (22.1)	13 (17.8)	
Recorded gestational age—days (mean [SD])	211.95 (39.54)	181.04 (13.60)	**<0.001**

*Note: p* values were calculated using a chi‐square test for categorical variables (with continuity correction) and ANOVA for continuous variables. *p* values below 0.05 are bolded.

### 3.2 Bronchoscopies

A total of 271 bronchoscopies were included in the study, with 175 (64.6%) performed in patients without BPD and 96 (35.4%) in those with BPD (As shown in Table 2). The average calendar year in which the bronchoscopy was performed was similar between groups (2022 ± 2.2 years in non‐BPD vs. 2022 ± 2.5 years in BPD; *p* = 0.986). Most bronchoscopies were performed in intubated patients (98.3% vs. 99.0%; *p* = 1.000), and trans nasal approaches were infrequent in both groups (2.3% vs. 2.1%; *p* = 1.000).

**Table 2:** Clinical and microbiologic characteristics of bronchoscopies by BPD status.

	No BPD	BPD	*p* value
Number of bronchoscopies	175	96	
Calendar year of bronchoscopy (mean [SD])	2022 (2.19)	2022 (2.49)	0.986
Sex = male (%)	111 (63.4)	58 (60.4)	0.72
Recorded race (%)			0.091
Asian	7 (4.0)	0 (0.0)	
Black or African American	71 (40.6)	50 (52.1)	
White	58 (33.1)	26 (27.1)	
Other or not recorded	39 (22.3)	20 (20.8)	
Age at time of bronchoscopy—days (mean [SD])	138 (68)	154 (53)	0.052
Trans nasal approach (%)	4 (2.3)	2 (2.1)	1
Patient intubated before bronchoscopy (%)	172 (98.3)	95 (99.0)	1
Length of intubation before bronchoscopy—days (mean [SD])	43 (50)	43 (34)	0.994
Time from bronchoscopy to extubation—days (mean [SD])	112 (165)	118 (139)	0.794
Respiratory severity score (mean [SD])	7.05 (4.08)	8.67 (4.13)	**0.003**
FiO2 on day of bronchoscopy (mean [SD])	40.60 (17.10)	44.95 (17.57)	0.053
Mean airway pressure on day of bronchoscopy (mean [SD])	16.71 (4.00)	18.97 (3.76)	**<0.001**
Broncho‐Alveolar lavage culture results			
Normal—(%)	45 (25.7)	11 (11.5)	**0.009**
*Escherichia coli*—(%)	11 (6.3)	8 (8.3)	0.702
*Proteus*—(%)	2 (1.1)	3 (3.1)	0.492
*Pseudomonas aeruginosa*—(%)	35 (20.0)	25 (26.0)	0.321
*Serratia marcescens*—(%)	18 (10.3)	7 (7.3)	0.552
*Staphylococcus aureus*—(%)	40 (22.9)	21 (21.9)	0.974
*Stenotrophomonas maltophilia*—(%)	25 (14.3)	15 (15.6)	0.906
*Moraxella catarrhalis*—(%)	0 (0.0)	2 (2.1)	0.240
*Klebsiella aerogenes*—(%)	5 (2.9)	0 (0.0)	0.230
*Klebsiella pneumoniae*—(%)	26 (14.9)	27 (28.1)	**0.013**
*Achromobacter species*—(%)	0 (0.0)	1 (1.0)	0.760
*Klebsiella oxytoca*—(%)	15 (8.6)	12 (12.5)	0.412
*Acinetobacter baumannii*—(%)	5 (2.9)	6 (6.2)	0.302
*Pluralibacter gergoviae*—(%)	1 (0.6)	0 (0.0)	1.000
*Citrobacter koseri*—(%)	1 (0.6)	2 (2.1)	0.596
*Enterobacter cloacae*—(%)	7 (4.0)	3 (3.1)	0.977
*Acinetobacter species*—(%)	2 (1.1)	0 (0.0)	0.757
Cell‐differential in broncho‐alveolar lavage			
Segmented Neutrophils—% (mean [SD])	66.98 (28.59)	71.48 (21.85)	0.524
Lymphocytes—% (mean [SD])	6.14 (7.91)	10.06 (6.24)	0.081
Monocytes—% (mean [SD])	6.79 (5.86)	5.88 (5.59)	0.613
Macrophages—% (mean [SD])	27.55 (30.50)	18.50 (21.86)	0.26
Eosinophils—% (mean [SD])	3.00 (4.20)	3.00 (3.37)	1

*Note:* This table summarizes demographic, clinical, and bronchoalveolar lavage (BAL) findings for 271 bronchoscopies performed in the neonatal intensive care unit (NICU), stratified by bronchopulmonary dysplasia (BPD) status. Continuous variables are presented as mean (standard deviation); categorical variables are presented as counts (percentages). *p* values were calculated using ANOVA for continuous variables and chi‐square tests with continuity correction for categorical variables. Statistically significant differences (*p* < 0.05) are bolded.

Patients with BPD were slightly older at the time of bronchoscopy, but this did not reach statistical significance (154.1 ± 53.5 vs. 138.3 ± 68.4 days; *p* = 0.052), a difference that potentially reflects the corrected age at which airway size permits placement of a 3.5 mm ETT and performance of bronchoscopy with BAL. Patients with BPD exhibited increased respiratory severity at time of bronchoscopy. Specifically, they had a higher RSS (8.67 vs. 7.05; *p* = 0.003) and higher MAP (18.97 vs. 16.71 cmH2O; *p* < 0.001) with differences in FiO2 not reaching significance (0.45 vs. 0.41; *p* = 0.053). Duration of intubation before bronchoscopy and time to extubation following the procedure were similar between groups.

**Figure 1:**
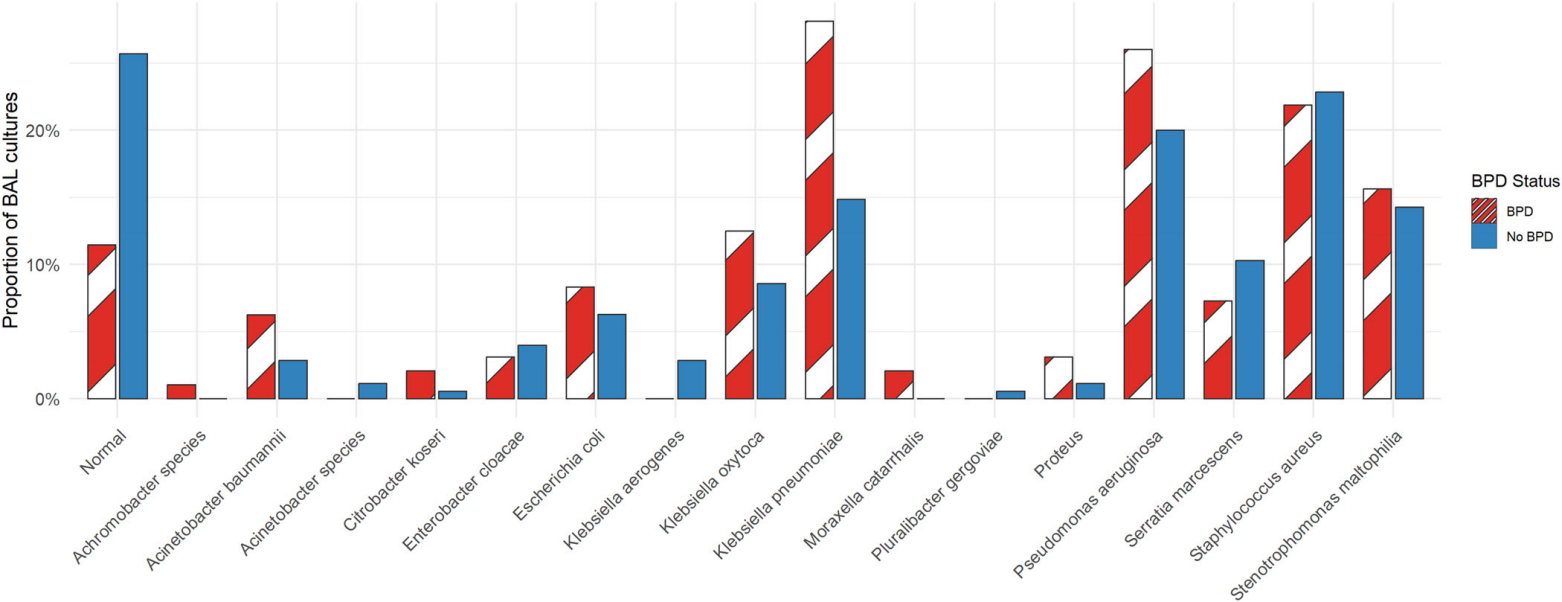
Proportion of bronchoscopies with each microbiologic finding, stratified by BPD status. This bar chart illustrates the distribution of microbiologic findings from bronchoscopic cultures among patients with (Red striped bars) and without (Blue bars) bronchopulmonary dysplasia. “Normal” indicates bronchoscopies in which either normal respiratory flora or no organisms were isolated. The presence of *Klebsiella pneumoniae* remained significantly associated with BPD in logistic regression models adjusted for sex and race, with robust standard errors clustered by patient (FDR‐adjusted *p* = 0.026). [Color figure can be viewed at wileyonlinelibrary.com]

BAL cultures were more frequently negative (i.e., classified as “Normal”) in the non‐BPD group (25.7% vs. 11.5%; *p* = 0.009, as shown in Figure 1). *K. Pneumoniae* was the only organism significantly more commonly identified in children later diagnosed with BPD (*p* = 0.013). Analysis of BAL fluid cell counts revealed no statistically significant differences in white blood cell differentials between groups.

### 3.3 Relationship **B**etween Culture Results and Clinical Outcomes

**Figure 2:**
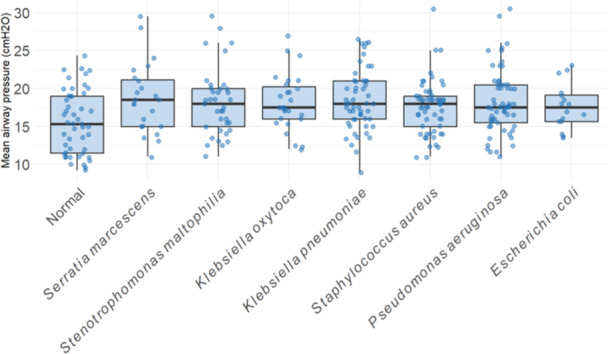
Respiratory severity score (RSS) by bronchoalveolar lavage (BAL) culture result. This figure shows the distribution of RSS on the day of bronchoscopy (preceding bronchoscopy), stratified by BAL culture findings. Each point represents an individual bronchoscopy; boxplots display the interquartile range (IQR) and median, with whiskers extending to ×1.5 IQR. [Color figure can be viewed at wileyonlinelibrary.com]

To examine the relationship between respiratory culture findings and disease severity, RSS were compared between bronchoscopies with normal cultures and those with positive cultures for common bacterial pathogens. A separate linear regression model was constructed for each bacterium, with RSS as the outcome and the organism's presence (vs. normal culture) as the exposure of interest. All models were adjusted for sex and race, and robust standard errors were used to account for repeated bronchoscopies within patients. there was no significant association between RSS and culture result. As a secondary analysis, we examined the relationship between culture result and MAP using the same covariates. After FDR correction the presence of *Escherichia coli* was associated with a significant increase in MAP (*β* = 2.28, FDR‐adjusted *p* = 0.047, Figure 2), as was *Pseudomonas aeruginosa* (*β* = 2.38, FDR‐adjusted *p* = 0.017), *Serratia marcescens* (*β* = 3.80, FDR‐adjusted *p* = 0.014), *Staphylococcus aureus* (*β* = 2.09, FDR‐adjusted *p* = 0.017), *Stenotrophomonas maltophilia* (*β* = 2.73, FDR‐adjusted *p* = 0.018), *Klebsiella oxytoca* (*β* = 2.53, FDR‐adjusted *p* = 0.017), and *K. pneumoniae* (*β* = 2.66, FDR‐adjusted *p* = 0.014). We next examined whether positive BAL cultures were associated with time to extubation following bronchoscopy. Separate negative binomial regression models were constructed for each organism, using days from bronchoscopy to extubation as the outcome, and adjusted for sex and race. Robust standard errors were used to account for clustering at the patient level. After FDR correction, none of the organisms tested were significantly associated with time to extubation (all FDR‐adjusted *p* > 0.83).

Finally, we assessed whether positive cultures were associated with a diagnosis of BPD. Logistic regression models were constructed separately for each organism, with BPD as the outcome and culture positivity as the exposure of interest. All models were adjusted for sex and race, and robust standard errors accounted for clustering by patient. After FDR correction, only *K. pneumoniae* remained significantly associated with BPD (FDR‐adjusted *p* = 0.026).

Given this association between *K. pneumoniae* and BPD and since MAP has a known association with BPD, we next examined whether the observed pathogen effects reflected underlying susceptibility to BPD or represented an independent contribution of bacterial colonization to elevated MAP. To this end, we repeated the model examening the relationship between *K. pneumoniae* positivity and MAP, now including BPD as a covariate. The association between *K. pneumoniae* and higher MAP remained significant (FDR‐adjusted *p* = 0.016), suggesting that the effect of *K. pneumoniae* on respiratory morbidity is not fully explained by a propensity to develop BPD.

### 3.4 Cell Counts Associated With *K. pneumoniae* Positive Cultures

For bronchoscopies with positive *K. pneumoniae* cultures, we compared BAL fluid cell counts between those with and without BPD to assess whether cellular profiles differed in ways that might suggest clinical relevance (as shown in Table 3). No differences in cell counts reached statistical significance.

**Table 3:** Differential cell counts in BAL fluid for *Klebsiella pneumoniae*‐positive BAL cultures, stratified by BPD status.

	No BPD	BPD	*p* value
Segmented Neutrophils—% (mean [SD])	63.30 (36.78)	75.62 (27.86)	0.445
Lymphocytes—% (mean [SD])	4.90 (4.18)	10.00 (8.12)	0.116
Monocytes—% (mean [SD])	5.86 (5.90)	4.20 (2.05)	0.565
Macrophages—% (mean [SD])	39.44 (42.02)	15.57 (24.28)	0.204
Eosinophils—% (mean [SD])	9.00 (9.90)	1.50 (0.71)	0.397

*Note:* Mean (standard deviation) percentages of immune cell subtypes from bronchoalveolar lavage (BAL) fluid are shown for patients with and without a diagnosis of bronchopulmonary dysplasia (BPD). *p* values were calculated using analysis of variance (ANOVA). None of the differences reached statistical significance.

## 4 Discussion

In this retrospective study of neonates undergoing flexible bronchoscopy with quantitative BAL culture, we found that several bacterial species were significantly associated with increased respiratory morbidity, as measured by MAP. In addition, *K. pneumoniae* was independently associated with a diagnosis of BPD. Notably, none of the bacterial species examined was significantly associated with prolonged time to extubation following bronchoscopy. These findings suggest that specific bacterial pathogens may contribute to heightened acute respiratory illness severity in preterm infants and may influence longer‐term pulmonary outcomes such as BPD.

These results are particularly important in the context of a longstanding knowledge gap: whether clinically available pulmonary microbiologic data, such as quantitative culture results, can meaningfully reflect disease severity or predict outcomes in this vulnerable population. Our study provides new evidence that quantitative BAL cultures, readily obtainable using modern flexible bronchoscopes, can identify bacterial species associated with both acute respiratory dysfunction and chronic lung disease. These findings help bridge the gap between microbiologic surveillance and actionable clinical information in neonatal respiratory disease. Although our study was limited to bronchoscopic specimens, these findings may also help inform the interpretation of pathogens identified from tracheal aspirates or ETT samples in acutely ill children.

While the retrospective design of our study limits the ability to draw causal inferences, our findings are consistent with prior reports demonstrating increased abundance of *Klebsiella* species in infants who go on to develop BPD, as well as higher prevalence of *K. pneumoniae* in those with more severe disease [9, 16]. The clinical relevance of this finding is further underscored by the recent emergence of hypervirulent *K. pneumoniae* strains. Although these strains currently tend to remain susceptible to standard antibiotics, there is growing concern about converging virulence and resistance traits, with some isolates now capable of producing carbapenemases and extended‐spectrum β‐lactamases [10, 17, 18]. The potential for such organisms to establish persistent colonization in the NICU environment and exacerbate respiratory morbidity in preterm infants warrants close attention.

Interestingly, we did not find significant differences in BAL fluid cell differentials between *K. pneumoniae*‐positive cultures from infants who did and did not go on to develop BPD. This finding is consistent with a previous study examining the airway microbiome of intubated neonatal patients that found no statistically significant differences in mean concentrations of cytokines, lipoteichoic acid, and lipo‐polysaccharide between the infants with and without BPD [9]. The lack of a statistically significant difference also cautions against overinterpreting relatively unremarkable cell counts as evidence of clinical insignificance. It is worth noting, however, that nearly all samples, regardless of BPD status, demonstrated a marked neutrophilic predominance. This overall pattern may have limited our ability to detect more subtle immunologic differences and highlights the need for additional biomarkers that better capture the clinical relevance of specific pathogens in this population.

Several limitations of this study should be considered when interpreting our findings. First, the retrospective design limits our ability to establish causality and introduces the possibility of unmeasured confounding. While we adjusted for sex and race in all models and clustered by patient, additional factors such as antibiotic exposure, underlying comorbidities, or variations in clinical management may have influenced both culture results and outcomes. Second, our reliance on culture‐based techniques, while reflective of real‐world clinical practice, may underestimate the presence of fastidious or unculturable organisms, and does not capture microbial diversity or community dynamics in the same way that sequencing‐based approaches do. Third, although we used a standardized threshold (≥10,000 CFU/mL) to define a positive culture, this cutoff does not necessarily indicate pathogenicity in all clinical contexts, and some lower‐level colonization may still be clinically relevant. Fourth, BAL cell counts were available only for a subset of patients, and the high prevalence of neutrophilic inflammation across the cohort may have limited our ability to detect meaningful differences in cellular composition. Finally, while RSS was our prespecified outcome, additional safeguards to avoid procedure‐related bias revealed higher variability of the FiO_2_ component, leading us to also examine MAP separately. We report this secondary analyses transparently, but acknowledge that it was performed post hoc.

In summary, this study demonstrates that specific organisms identified on quantitative BAL culture are associated with increased respiratory severity in intubated neonates, and that *K. pneumoniae* in particular may also be linked to the development of BPD. These findings support a growing body of literature implicating dysbiosis and opportunistic pathogens in the pathogenesis of neonatal lung disease and underscore the potential value of incorporating quantitative microbiologic data into risk stratification frameworks. As neonatal respiratory care continues to evolve, prospective studies that integrate culture results with molecular profiling, host immune markers, and longitudinal clinical outcomes will be critical for identifying modifiable contributors to disease progression and improving precision in the management of respiratory illness in preterm infants.

## Author Contributions

**Jelte Kelchtermans:** conceptualization, methodology, formal analysis, visualization, writing – original draft, investigation. **Pelton Phinizy:** conceptualization, writing – review and editing, methodology. **Joseph Piccione:** conceptualization, writing – review and editing, supervision, methodology. **Sharon A. McGrath‐Morrow:** conceptualization, methodology, writing – review and editing, supervision. All authors approved the final manuscript.

## Ethics Statement

This study protocol was reviewed and classified as Exempt from IRB review and informed consent under category 4 by the CHOP IRB (IRB protocol 24‐022076).

## Conflicts of Interest

The authors declare no conflicts of interest.

## Data Availability

The data that support the findings of this study are available from the corresponding author, J.K., upon reasonable request.
